# Enhancing Osteogenic Potential: Controlled Release of Dopamine D1 Receptor Agonist SKF38393 Compared to Free Administration

**DOI:** 10.3390/biomedicines12051046

**Published:** 2024-05-09

**Authors:** Yunwei Hua, Chenxi Wang, Xiyuan Ge, Ye Lin

**Affiliations:** Department of Implantology, Peking University School and Hospital of Stomatology & National Clinical Research Center for Oral Diseases & National Engineering Laboratory for Digital and Material Technology of Stomatology & Beijing Key Laboratory of Digital Stomatology, Beijing 100081, China; hyw_0226@163.com (Y.H.); wcx-0604@163.com (C.W.)

**Keywords:** osteogenic, hBMSCs, osteoporosis, PLGA, dopamine receptor, SKF38393

## Abstract

Osteoporosis is the most common metabolic bone disorder and is characterized by decreased bone density, which has a relationship with the quality of life among the aging population. Previous research has found that activation of the dopamine D1 receptor can improve bone mass formation. SKF38393 is an agonist of dopamine D1 receptors. However, as a small-molecule drug, SKF38393 is unstable and releases quickly. The aim of this study was to prototype polylactic-co-glycolic acid (PLGA)/SKF38393 microspheres and assess their potential osteogenic effects compared to those under the free administration of SKF38393. The cytocompatibility of PLGA/SKF38393 was determined via CCK-8 and live/dead cell staining; the osteogenic effects in vitro were determined with ALP and alizarin red staining, qRT-PCR, and Western blotting; and the in vivo effects were assessed using 25 Balb/c mice. We also used a PCR array to explore the possible signaling pathway changes after employing PLGA/SKF38393. Our experiments demonstrated that the osteogenic effect of D1Rs activated by the PLGA/SKF38393 microsphere was better than that under free administration, both in vitro and in vivo. According to the PCR array, this result might be associated with six signaling pathways (graphical abstract). Ultimately, in this study, we prototyped PLGA/SKF38393, demonstrated its effectiveness, and preliminarily analyzed its mechanism of action.

## 1. Introduction

Osteoporosis is a condition involving decreased bone mass and increased bone fragility. This disease is believed to be caused by the destruction of bone homeostasis and is characterized by the destabilized equilibrium of osteoblastic and osteoclastic processes, which inhibits bone remodeling. About 40% of fractures around the hips, vertebrae, and wrists are associated with osteoporosis [1]. Moreover, the risk of osteoporosis in women is twice as high as that in men [2] due to the sudden drop in estrogen after menopause [3]. In recent years, the disease burden from osteoporosis has grown in multiple regions across the world due to the increasing age of the population. Yamazaki found that after implantation for 7 weeks, the bone–implant contact (BIC) in the tibia of ovariectomized (OVX) rats was significantly lower than that in the control groups. This phenomenon suggests that osteoporosis may influence the implant’s primary stability [4]. Li’s experiment also found osteoporosis to have destructive effects on previously osteo-integrated implants inserted into the distal metaphysis of the femur in SD rats [5]. In addition, previous studies established the association between the nervous system—specifically the sympathetic nervous system (SNS)—and bone formation [6] and that the excitatory molecules of SNS receptors, such as β2AR, can regulate osteoblasts and osteoclasts [7,8,9]. As a result, our research focuses on the regulation of nervous system receptors.

Dopamine (DA) has been researched as a neurotransmitter in several areas, such as neuroscience and psychological medicine [10]. DA can regulate many functions, such as emotion, addiction, reward, and sleep. In 2000, Bliziotes et al. found that knockout of the dopamine transporter (DAT) can reduce bone formation and strength in mice, with DAT−/−mice found to have lower cancellous bone volume, lesser ash calcium content, and higher trabecular space in their bones [11]. Subsequently, more studies found that DA can bind with the receptors on osteoblasts and affect bone remodeling and bone metabolism [12]. These previous studies confirmed the association between DA and bone formation and led to further research that established multiple pathways for DA’s effects on osteogenesis.

The DA receptor is a member of the G-protein coupled receptor (GPCR) family, which comprises seven transmembrane receptors [13]. This family also contains the parathyroid receptor and calcium-sensing receptor [14,15]. Many drugs target GPCRs and trigger various functions through different pathways. The DA receptor includes D1, D2, D3, D4, and D5 subtypes and can be divided into D1-like receptors (D1Rs) (D1 and D5) and D2-like receptors (D2Rs) (D2, D3, and D4) [16]. D1Rs can activate the Gs protein, while D2Rs can activate the Gi protein [17,18]. Wang et al. found that the activation of D1Rs can promote bone formation and enhance osteogenic differentiation in human bone marrow stem cells through the MAPK/ERK signaling pathway [19], and Feng et al. found that D1Rs can activate the Wnt signaling pathway to reduce the inhibition of osteogenesis [20]. These studies determined that D1Rs can influence bone homeostasis in both osteogenic and osteoclastic directions. Although the effect of D1Rs on bone formation was established in previous studies, the mechanism behind this effect remains unclear [21]. SKF38393 is an agonist of the D1R and was previously found to improve osteogenic abilities in human bone marrow mesenchymal stem cells (hBMSCs) [16].

However, as a small molecule, SKF38393 releases quickly and does not have long-lasting effects [22,23]. For this reason, global research is currently focused on determining how bone tissue engineering methods could extend the release time and promote the effects of SKF38393 and other small-molecule drugs. Polylactic-co-glycolic acid (PLGA) is a type of polymer organic compound that has been widely used in the pharmaceutical and medical engineering fields. The advantages of PLGA include a simple preparation method, affordable price, and stable effects [24,25]. In recent years, many researchers have used PLGA in bone-related studies to create composite materials such as PLGA/HA, PLGA/Mg, and PLGA/COL to enhance osteogenesis [26,27]. Cai’s research reported using PLGA-Icariin/Mg^2+^ to control the release of Icarlin and Mg^2+^, achieving release lasting more than 21 days with significant osteogenic impacts [28]. According to previous research, we hypothesized that PLGA might have the potential to ameliorate the defects of SKF38393, which always releases quickly. In this study, we prototyped PLGA/SKF38393 microspheres to determine if they have osteogenic effects and whether such effects are better than those under free admission of SKF38393.

## 2. Materials and Methods

### 2.1. Preparation of PLGA/SKF38393 Microspheres

**Figure 1:**
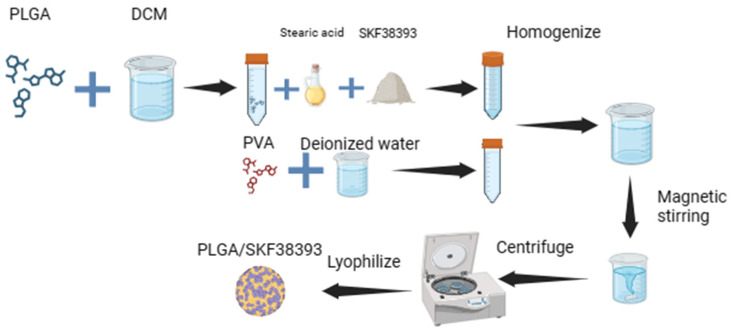
Infographic showing the fabrication of PLGA/SKF38393 via the S/O/W method. Created with Biorender.

PLGA/SKF38393 microspheres were created using the solid-in-oil-in-water (S/O/W) method, as shown in Figure 1. In total, 300 mg of PLGA was dissolved in 3 mL of dichloromethane (DCM). Then, we added 20 mg of stearic acid and 50 mg of SKF38393 to the mixture and used ultrasound to turn the mixture into a homogenous solution, referred to as solution A. Next, we dissolved 100 mg of polyvinyl alcohol (PVA) in 50 mL of deionized water, which was labeled solution B. We mixed and homogenized solution A with solution B by stirring the solution with a magnetic mixer. After volatilization for 5 h, the microspheres were collected via centrifugation, washed with deionized water three times, and lyophilized.

### 2.2. Physicochemical Property Analyses of PLGA/SKF38393 Microspheres

Scanning electron microscopy (SEM) (S-3000N, Hitachi, Düsseldorf, Germany) was employed to observe the surfaces of microspheres with or without SKF38393. A Fourier transform infrared (FTIR) spectrometer (Nicolet IS10, Thermo Fisher Scientific, Waltham, MA, USA) was used to perform FTIR spectroscopy on the microspheres. The chemical compositions and changes in the chemical bonds in the coatings were detected after natural drying. We collected each spectrum from 400 to 4000 cm^−1^ at a resolution of 0.4 cm^−1^ with 25 scans.

### 2.3. SKF38393 Release Profile Testing Using High-Performance Liquid Chromatography (HPLC)

High-performance liquid chromatography (HPLC) was used to measure the release of SKF38393 from the microspheres with an Xselect T3 column (250 × 4.6 mm, 5 μm, Waters, Milford, MA, USA). A 0.1% phosphoric acid/acetonitrile (9:1) solution was used as the mobile phase, and over 3000 theoretical plates were applied at a flow rate of 0.8 mL/min and a detection wavelength of 280 nm. The column temperature was set at 35 °C, and the injection volume was 15 μL. The dialysis bag method was applied to assess the in vitro release of SKF38393 from the microspheres. Dialysis bags (3.5 kDa) (spectrum labs, Los Angeles, CA, USA) were cut to a suitable volume, filled with 5 mL of PLGA/SKF38393, and placed in 50 mL of PBS buffer in a flask. The release of SKF38393 from the PLGA/SKF38393 microspheres was analyzed at 37 °C in PBS buffer at a constant pH value (pH 7.4). One milliliter of the release media was obtained at each indicated time point, and its SKF38393 content was measured. Simultaneously, 1 mL of PBS buffer was added to the release media to maintain the same environment. External media were replaced every other day, at which time the data were collected to calculate the cumulative release amount and draw the release curve. The encapsulation efficiency test was carried out as follows. The actual drug load of the microspheres was calculated using a standard curve, and the encapsulation efficiency was calculated (actual drug load of the microspheres)/(total drugs used).

### 2.4. Cell Culture and Osteogenic Differentiation

Next, the hBMSCs were purchased as cryopreserved frozen cells (Cyagen Biosciences Technology, Guangzhou, China). The cells were cultured with 10% fetal bovine serum (FBS) (Gibco, New York, NY, USA) and 1% penicillin/streptomycin at 37 °C. The atmosphere was kept humid at 95% air, and 5% CO_2_ with a culture medium refreshed every 2 days. To stimulate osteogenic differentiation, we seeded the BMSCs onto a 6-well plate at a density of 40,000 cells per mL and a 12-well plate at 20,000 cells per mL. When 80–90% confluence was observed in the cells, we replaced the culture medium with an osteogenic medium (OriCell™ Human MSC Osteogenic Differentiation Medium, Cyagen Biosciences, Guangzhou, China), which was changed after 3–4 days.

### 2.5. Cell Proliferation

Then, the hBMSCs were plated into a 96-well dish with 15,000 cells and divided into 4 groups as follows: 0 μmol/L, 1 μmol/L, 10 μmol/L, and 100 μmol/L of SKF38393. These concentration levels were chosen based on the findings from previous studies [13]. For the PLGA/SKF38393 groups, the concentration of SKF38393 was calculated via the drug loading ratio on days 1, 3, and 7, and the microspheres were simultaneously provided. After 1, 3, and 7 days, the culture medium in each well was replaced with 110 μL of a 10% CCK-8 buffer assay (Dojindo, Kyushu, Japan) and incubated at 37 °C for 2 h. The optical density (OD) of each well was detected using an ELX-808 absorbance microplate recorder (BioTek, Winooski, VT, USA) at 450 nm. The average value of each group was used for the result analysis, and this experiment was repeated independently three times with SKF38393 and then with PLGA/SKF38393 at 0 μmol/L, 1 μmol/L, 5 μmol/L, and 10 μmol/L.

### 2.6. Cytocompatibility and Cell Adhesion

To observe the effects of PLGA/SKF38393 on cell adhesion, the cells were plated on a confocal dish with 30,000 cells/dish, cultured with an osteogenic induction medium (HUXXC-90021, Cyagen, Guangzhou, China) for 7 days, and then fixed with paraformaldehyde (4%) for 30 min. Next, the cells were stained with 0.1% phalloidin (Abcam, Cambridge, UK) and ProLong^®^ Gold Antifade Reagent with 4′,6-diamidino-2-phenylindole (DAPI) (CST, Danvers, MA, USA) according to the manufacturer’s instructions. Immunofluorescence images were captured with a confocal microscope (FV3000, Olympus, Tokyo, Japan), and the safety of PLGA/SKF38393 in hBMSCs was confirmed via live/dead cell staining.

### 2.7. Alkaline Phosphatase (ALP) Activity Assay, ALP, and Alizarin Red Staining

The hBMSCs were divided into 3 groups: the control group, the SKF38393 group, and the PLGA/SKF38393 group. The hBMSCs were seeded into 6-well plates at a density of 80,000 cells per well. When the cell density reached 80%, the culture medium was changed to an osteogenic medium, and PLGA/SKF38383 microspheres were added to the plates with the medium. After 7 days of osteogenic differentiation, the samples were washed with a PBS solution 3 times at room temperature. The cells were then fixed in 4% paraformaldehyde for 30 min and stained with a 5-bromo-4-chloro-3-indolyl-phosphate (BCIP)/nitro blue tetrazolium (NBT) Alkaline Phosphatase Color Development Kit (Beyotime Institute of Biotechnology, Shanghai, China) for 20 min. Next, we washed the cells several times with PBS and analyzed them via microscopy. The ALP activity was measured using an ALP activity kit (JianCheng Bioengineering Institute, Shanghai, China). The results were normalized based on the levels of total protein and measured with the BCA method (Thermo Fisher Scientific, Waltham, MA, USA). Alizarin red staining was applied to hBMSCs after 14 days of osteogenic differentiation, and all samples were washed with a PBS solution 3 times and fixed in 95% ethyl alcohol for 20 min. After washing the wells 3 times, the cells were analyzed under a microscope.

### 2.8. Quantitative Real-Time PCR (q-PCR)

The total mRNA from the cells was seeded onto a 6-well plate at a density of 80,000 cells per well and extracted using a cell RNA extract kit (Foregene, Chengdu, China). The mRNA was quantified via UV spectrophotometry, and only samples with a ratio of absorbance at 260 and 280 nm (the 260/280 ratio) between 1.8 and 2.1 were used in the following steps. The total mRNA was reserve-transcribed into cDNA using a PrimeScript RT Reagent Kit (TaKaRa, Gunma, Japan). FastStart Universal SYBR Green Master Mix (Roche, Basel, Switzerland) was mixed with the cDNA, and q-PCR was performed by applying Q3 (Applied Biosystems, Thermo Fisher Scientific, Waltham, MA, USA). Relative quantization was determined via the ΔΔCt method, and the housekeeping gene glyceraldehyde 3-phosphate dehydrogenase (GAPDH) was used for normalization. The following sequences of gene primers were used for q-PCR: RUNX2 (forward 5′-CCATAACGGTCTTCACAAATCCT-3′and reverse 5′-TCTGTCTGTGCCTTCTTGGTTC-3′), ALP (forward 5′-CTGGTACTCAGACAACGAGATG-3′ and reverse 5′-GTCAATGTCCCTGATGTTATGC-3′), and GAPDH (forward 5′-GAGTCCACTGGCGTCTTCAC3′ and reverse 5′-TTCACACCCATGACGAACAT-3′).

### 2.9. Protein Extraction and Western Blot Analysis

The total proteins from hBMSCs were extracted through lysis in a radioimmunoprecipitation assay (RIPA) buffer containing a protease inhibitor cocktail (Solarbio, Beijing China). BCA protein kits (Thermo Fisher Scientific, Waltham, MA, USA) were used to quantify the proteins in each group. About 20 µg of protein mixed with the loading buffer (Solarbio, Beijing, China) was separated using 10% Tris-glycine sodium dodecyl sulfate-polyacrylamide gel electrophoresis (SDS-PAGE). Then, the proteins were transferred onto a polyvinylidene fluoride membrane for immunoblotting. We used 5% skim milk in Tris-buffered saline and a Tween 20 (TBST) buffer for blocking; then, the membrane was incubated for one hour at room temperature. After blocking, the membrane was incubated with the primary antibodies against rabbit Runx2 (CST, Danvers, MA, USA), ALP, and GAPDH (Abclonal, Wuhan, China) overnight at 4 °C. The membrane was washed with TBST three times, and horseradish peroxidase-linked secondary antibodies (Abclonal, China) were used to detect the primary antibodies. The secondary antibodies were incubated for 1 h at room temperature. Both the primary antibody and secondary antibodies were diluted using an antibody dilution buffer (New cell and Molecular Biotech, Suzhou, China) according to the instructions (Runx2 1:1000; ALP 1:1000; GAPDH 1:5000; secondary antibodies 1:10,000). After washing the membranes three times, an enhanced chemiluminescence (ECL) reagent (Abclonal, Wuhan, China) was used to improve visualization under the e-blot (New Prolife, Beijing, China), and images were used to measure the gray values of all target proteins with ImageJ software (National Institutes of Health, Bethesda, MD, USA).

### 2.10. Animal Experiments

Twenty-five 12-week-old female Balb/c mice weighing about 20 g were used in this experiment. The animals were divided into 5 groups: the CTL group, the sham group, the OVX group, the SKF38393 group, and the PLGA/SKF38393 group. The animal experiments were approved by the Institutional Animal Care and Use Committee of Peking University Health Science Center (No. LA2022235). The animals were cared for in accordance with international standards and also conformed with the requirements of the Animal Research Committee of Peking University. The mice were bought and seeded in the Animal Experimental Center of the Peking University Health Science Center. Five mice were placed in each cage and kept under climate-controlled conditions with a temperature of 22–24 °C, 50–60% humidity, and a 12-h light/dark cycle. All mice had the same diet and free access to standard food (12% calories) and water. After 1 week of acclimation, the OVX group, SKF38393 group, and PLGA/SKF38393 group received OVX operations, while the mice in the sham group received sham operations in which their abdominal cavities were exposed and sutured, but no internal organs were removed. After the operation, mice received antibiotic injections for 3 days, based on experience from previous studies [29]. The SKF38393 group had an intraperitoneal injection of 10 mg/kg SKF38393 (MCE, Shanghai, China) every day, and the PLGA/SKF38393 group was injected with the PLGA/SKF38393 microspheres using the same dosage of SKF38393 applied in the SKF38393 group. The microspheres were dissolved in saline and injected with a 1 mL injection syringe. The other two groups were injected with the same volume of saline. After 8 weeks, the mice were euthanized.

### 2.11. Micro-CT

Bone samples from the femur were used for the analysis and histological testing. All samples were fixed in a 4% concentration of paraformaldehyde and scanned using a SkyScan 1176 (Bruker skyscan, Antwerp, Beligum). Images were reconstructed with a voxel size of 10 μm. The scanning system was set to an integration time of 70 kV, 114 μA, and 700 ms, and a three-dimensional Gaussian filter (sigma = 1.2, support = 2) was used to eliminate the noise in the volumes. The region of interest (ROI) included a 1 mm thick portion of the trabecular bone from the tibia in the axial plane below the growth plate (Supplementary Figure S1). In addition, the BMD, percentage of bone volume ratio (BV/TV, %), trabecular separation (Tb.Sp, mm), and trabecular thickness (Tb.Th, mm) were used to analyze the bone mass.

### 2.12. Histologic Analysis

The mice were euthanized 8 weeks after the OVX operation, and each group received the same treatment used for Micro-CT. All samples were decalcified and curetted with a Leica CM3050S (Leica Microsystems AG, Wetzlar, Germany). The samples were sectioned vertically through the maximum cross-section, with slices about 3 μm thick. Afterward, the samples were stained with hematoxylin and eosin (HE) and Masson. All images were observed and captured with a Zeiss Imager Z2 microscope (Zeiss, Jena, Germany).

### 2.13. Osteogenic PCR Array

The RNA was extracted from the hBMSCs and reverse transcribed to cDNA, mixed with qPCR SYBR, added into the 96-well plate osteogenesis PCR array (WcGENE Biotech, wc-mRNA0202-M, Shanghai, China), and subjected to RT-qPCR. Gene expression was calculated as 2^−∆∆Ct^ relative to Actb as the endogenous control.

### 2.14. Statistical Analysis

IBM SPSS Statistics for Windows, Version 19.0 (IBM, Armonk, NY, USA) was used to compare the results between all groups. A one-way ANOVA followed by Newman–Keuls post hoc tests was used to determine significant differences with the same homoscedasticity. Moreover, *p* < 0.05 and *p* < 0.01 were considered to indicate significant differences.

## 3. Results

### 3.1. Synthesis and Characterization of PLGA/SKF38393 Microspheres

**Figure 2:**
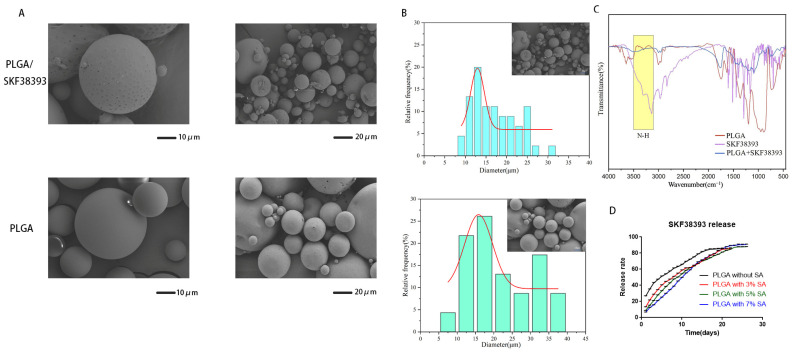
(**A**) SEM images of PLGA and PLGA/SKF38393. (**B**) Particle size analysis of PLGA and PLGA/SKF38393. (**C**) FT-IR spectra of PLGA, SKF38393, and PLGA/SKF38393. (**D**) Release rate of SKF38393 in PLGA/SKF38393.

As shown from the scanning electron microscopy (SEM) results, the diameter of PLGA and PLGA/SKF38393 ranged from about 10 μm to 80 μm. We chose a screening area with more than 10 uniform microspheres to generate images for particle size analysis. The morphology of the microspheres lacked obvious broken debris, and the average diameters were about 22.02 µm. The average diameters of the PLGA/SKF38393 microspheres were 17.13 µm and featured rougher surfaces, possibly due to the encapsulation of inorganic powders. Then, the chemical bonds in SKF38393 were determined, with the corresponding Fourier transform infrared spectroscopy (FT-IR) spectra provided in Figure 2. As also shown in Figure 2, the characteristic peaks determined for independent component analysis (ICA) at 3400 cm were compared with those using the PLGA/SKF38393 to confirm that SKF38393 had mixed with PLGA.

We used stearic acid to stabilize the release of SKF38393 and reduce the burst release of this molecularly small drug. Then, we tested the microsphere release profiles with different concentrations of stearic acid. The HPLC results showed that the burst release (day 01) decreased from 25% to 13%, 9%, and 7%, respectively, with 3% (*w*/*w*), 5% (*w*/*w*), and 7% (*w*/*w*) stearic acid. Each group showed a controllable release of SKF38393 over 20 days, with both the 5% and 7% groups continuing to release the compound for 26 days. The group with 5% (*w*/*w*) stearic acid presented the highest drug-loading (16.2%) and encapsulation rates (87.4%), while the release rate also achieved about 88%. We used this parameter for subsequent research.

### 3.2. Cytocompatibility, Cell Adhesion, and Cell Proliferation

**Figure 3:**
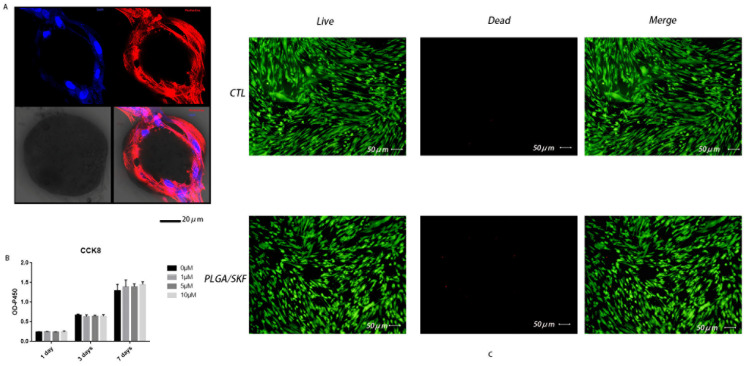
(**A**) Fluorescent staining indicating the cell adhesion of PLGA/SKF38393. (**B**) The safe concentration of PLGA/SKF38393 based on a CCK-8 assay. (**C**) Live/dead staining.

We applied a concentration-response experiment to determine the safe concentration of SKF38393 in hBMSCs using a cell counting kit-8 assay (Supplementary Figure S2). Our results showed that using 10 μmol/L SKF38393 or PLGA/SKF38393 (10 μmol/L SKF38393) had little influence on the proliferation of hBMSCs (Figure 1). Thus, we decided to use 10 μmol/L as the concentration of SKF38393 in this experiment. Cell adhesion was determined via the immunofluorescent staining of phalloidin. Ultimately, we observed a satisfactory cell adhesion level for PLGA/SKF38393. Live/dead staining also showed that PLGA/SKF38393 had decent cytocompatibility in hBMSCs. These results are all presented in Figure 3.

### 3.3. SKF38393 and PLGA/SKF38393 Regulate the Differentiation of hBMSCs

**Figure 4:**
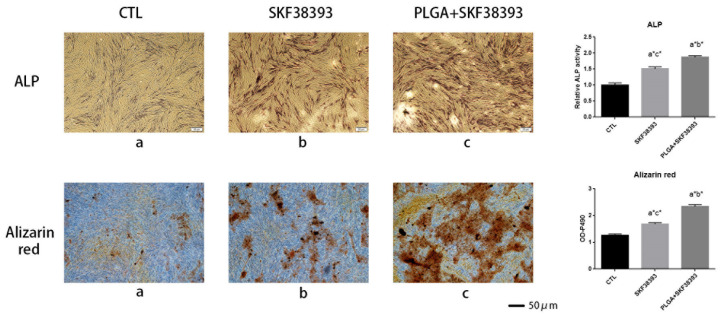
ALP staining and relative ALP activity: Alizarin red staining and total absorbance measurements during late hBMSC osteogenic differentiation stimulated with SKF38393 and PLGA/SKF38393 (*n* = 3 for all groups), a: CTL; b: SKF38393; c: PLGA+SKF38393. * *p* < 0.05.

**Figure 5:**
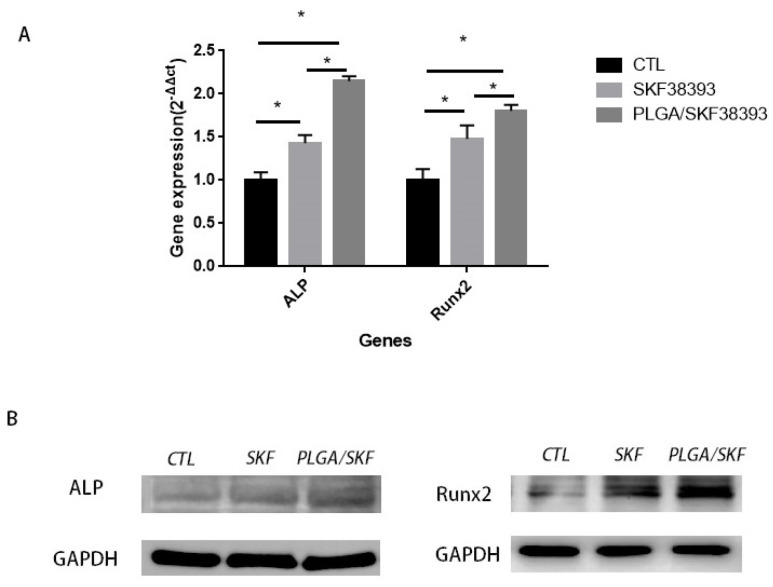
(**A**) Quantitative RT-PCR analysis of ALP and Runx2 expression during hBMSC osteogenic differentiation stimulated with SKF38393 and PLGA/SKF38393 (*n* = 3 for all groups), * *p* < 0.05. (**B**) Western blot analysis of ALP and Runx2 expression during hBMSC osteogenic differentiation stimulated with SKF38393 and PLGA/SKF38393.

The early differentiation of hBMSCs was investigated via ALP staining and ALP activity assays, while the final mineralization of hBMSCs was determined using ARS staining. Both ALP and ARS staining showed that using SKF38393 and PLGA/SKF38393 had a positive effect on osteogenesis, while the influence of the PLGA/SKF38393 group was slightly better than that of the SKF38393 group (Figure 4). In these groups, the gene expression of ALP and Runx2 at 7 days after the activation of the D1R also showed a similar tendency. At the protein level, the expression of ALP and Runx2 was measured at 7 days. These markers indicated that the activation of the D1R can promote osteogenesis, with the use of PLGA/SKF38393 found to be better than the free addition of SKF38393 (Figure 5). We also found, with q-PCR, that using only PLGA had no effect on osteogenesis, which indicates that the PLGA material does not have an osteogenic effect and that the effect from the PLGA/SKF38393 group was solely a product of SKF38393. As a result, the PLGA group was not used in additional experiments (Supplementary Figure S3).

### 3.4. Animal Experiments

**Figure 6:**
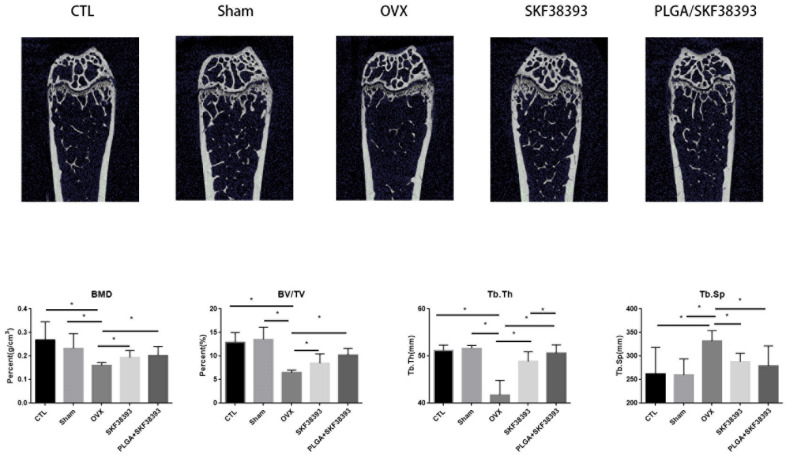
Micro-CT images of the ROIs and changes in the BV/TV, Tb.th, Tb.Sp, and BMD of the femurs in all five groups. The parameters are expressed as the mean ± SD, *n* = 5 specimens per group, * *p* < 0.05.

**Figure 7:**
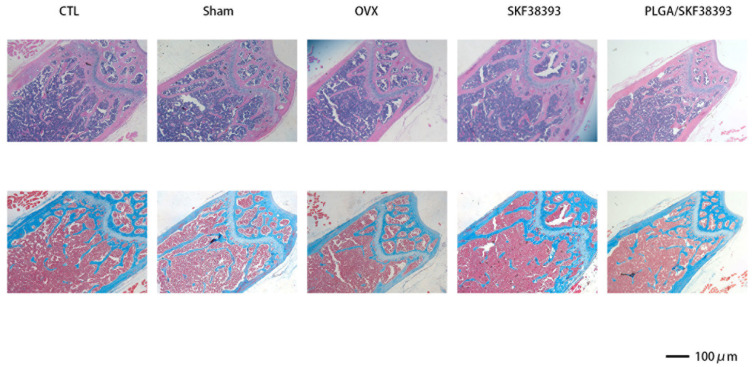
Changes in the histological test for all five groups determined via HE staining and Masson staining.

The femurs used in our experiment showed significant changes after the intraperitoneal injection of SKF38393 and PLGA/SKF38393 compared with the results in the CTL group. In the micro-CT analysis, we found that the parameters of BMD, BV/TV, and Tb.Th increased significantly in the PLGA/SKF38393 group and the SKF38393 group, while Tb.Sp decreased in these two groups. Additionally, the osteogenesis effect of PLGA/SKF38393 was slightly better than that in the SKF38393 group (Figure 6). After micro-CT scanning, the samples were used for histological analysis, the results of which were similar to those of the micro-CT. HE staining and Masson staining showed that both SKF38393 and PLGA/SKF38393 can enhance osteogenesis and that PLGA/SKF38393 offered a better effect than SKF38393 alone (Figure 7).

### 3.5. Different Effects of SKF38393 and PLGA/SKF38393 on Osteogenesis in the PCR Array

**Figure 8:**
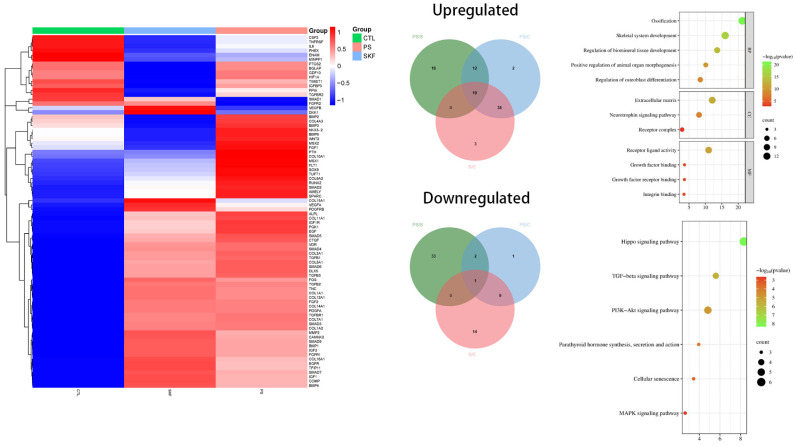
The q-PCR array results showing the mRNA expression involved in osteogenic differentiation in hBMSCs stimulated with SKF38393 and PLGA/SKF38393.

We observed 36 upregulated genes and 40 downregulated genes in the hBMSCs grown on PLGA/SKF38393. The upregulated genes contained osteoblast-differentiation-related genes such as Bone Morphogenetic Protein 2 (BMP2), Bone Gamma-carboxyglutamate (gla) Protein (BGLAP), and ALP. Upregulated transcription factors included Runt-related transcription factor 2 (RUNX2), transcription factor Sp7 (SP7), and SMAD family member 7 (SMAD7). Human cell adhesion and extracellular matrix (ECM) gene expression analyses showed upregulated factors such as Fibroblast Growth Factor 1 (FGF1) and Fibroblast Growth Factor 1 (FGF1). Moreover, some genes coding ECM molecules and adhesion molecules, such as Fms-Related Receptor Tyrosine Kinase 1 (FLT1), also showed upregulation tendencies. A GO analysis of these genetic changes indicated that using PLGA/SKF38393 could offer better osteogenesis effects through the Hippo pathway. The MAPK signaling pathway, Wnt signaling pathway, and TGF-beta signaling pathway were also associated with osteogenesis in this experiment (Figure 8).

## 4. Discussion

Osteoporosis has been a worldwide health issue for a long period of time. In the past decades, many relevant mechanisms have been discovered, and some drugs in other areas have been identified to treat osteoporosis. For example, while metformin is widely used in diabetic patients, it was also found to improve bone mass in rats with osteoporosis [30]. In addition, some anti-osteoporosis drugs were found to have a negative effect during treatment. For example, Marx et al. found that bisphosphonate drugs (BPs) can cause drug-induced necrosis of the jaw [31]. Another drug named calcitonin offers anti-osteoporosis effects by inhibiting the functions of osteoclasts [32], but some research also found that calcitonin inhibits the functions of both osteoblasts and osteoclasts and may decrease the body’s bone formation capabilities [33]. Another treatment for osteoporosis is estrogen replacement therapy. Liu’s research found that using Raloxifene may increase the risk of several gastroesophageal diseases, such as gastroesophageal reflux disease and Barrett’s esophagus in postmenopausal women with osteoporosis [34]. In recent years, finding new drugs for osteoporosis has become a key research area.

Dopamine and its derivatives were commonly studied as psychotropic medicines [35] in past bone-related research. Recent studies revealed that the activated D1R can promote osteogenic differentiation through several pathways. Zhu et al. found that the activation of the dopamine receptor D1 can reduce glucocorticoid-related bone loss by upregulating the ERK1/2 signaling pathway [36], while other studies found that the D1R could also enhance bone remodeling via the Wnt signaling pathway. Compared with the anti-osteoporosis effects of BPs and calcitonin, which inhibit bone formation and resorption, our research confirmed the activation of the dopamine D1 receptor to promote bone formation. However, as small-molecule drugs, DA and SKF38393 always suffer from performance instability, a short onset time, and harmful decomposition products. For these reasons, the effects of freely administered DA and SKF38393 do not remain stable for a long period of time. The controlled release of SKF38393 can not only offer a stable release rate of the drug but also prolong the duration of SKF38393. This method could also enhance the effects of SKF38393.

Many successful bone tissue engineering materials, including hydrogels, metal–organic frameworks (MOFs), and PLGA, have been used by previous scholars in bone regeneration and the development of controlled-release drugs [37,38,39]. Our team’s previous study found that using DA-loaded alginate–arginine–glycine–aspartic acid coatings on titanium surfaces can successfully promote bone remodeling [40]. Therefore, slow-release dopamine remains effective, which suggests a new avenue for extending the short-release time of dopamine. However, there are some disadvantages to these materials. For example, Yang’s research noted that MOF materials such as ZIF-8 produce short blood circulation and poor targeting, which can reduce drug efficiency [41]. Using a hydrogel also has some problems, such as poor biocompatibility and unusual in vivo biodegradation [42,43]. Consequently, we selected PLGA because of its good biocompatibility and superior sustained-release capabilities [44]. In this research, we used PLGA/SKF38393 microspheres to control the release of SKF38393. The results showed positive effects in both hBMSCs and mice with osteoporosis. Indeed, the application of PLGA/SKF38393 produced better bone formation comparison with the free administration of SKF38393. This result indicates that PLGA can combine well with SKF38393 and has potential in osteoporosis therapy.

We also conducted a genetic sequencing analysis and identified six possible signaling pathways that might be associated with this phenomenon. The Hippo signaling pathway showed the strongest association, which suggests that ECM plays an important role in enabling or modifying SKF38393′s effects on bone formation. Our immunofluorescence staining results confirmed this role and showed that cells adhered well around the microspheres. SKF38393 can likely upregulate the expression of ECMs such as COL10A1. Based on the GO and KEGG analyses, the MAPK and TGF-β signaling pathways were also enhanced with SKF38393.

Some limitations in our research should be acknowledged. The 8-week observation period used in this study was able to reflect early bone mass changes in osteoporosis in mice, but the longer-term drug effects remain unknown. Moreover, the physiological characteristics of OVX mice are distinct from those of human beings in a few key ways. Thus, future scholars should be cautious about generalizing the conclusions of our study to human subjects. Moreover, our sample size was too small to perform mechanical tests, which could be a focus of future research.

## 5. Conclusions

In our study, we successfully designed and fabricated a material for bone tissue engineering, PLGA/SKF38393, to control the release of the dopamine D1 receptor agonist SKF38393. The results showed satisfactory cytocompatibility. Compared with the free administration of SKF38393, PLGA/SKF38393 offered better osteogenic results in both hBMSCs and mice. A PCR array showed six signaling pathways to be associated with the osteogenic effects of PLGA/SKF38393, with the Hippo signaling pathway found to have the strongest correspondence. Our research determined a method to enhance the effects of SKF38393, and we preliminarily explored the corresponding mechanism. This method could also have great potential in osteoporosis therapy and improve the efficacy of other small-molecule drugs in additional areas.

## Data Availability

The data supporting this study are available from the corresponding author upon reasonable request.

## Supplementary Materials

The following supporting information can be downloaded at: https://www.mdpi.com/article/10.3390/biomedicines12051046/s1, Figure S1: ROI of Micro-CT; Figure S2: Safety concentration of SKF38393 by CCK-8 assay. * *p* < 0.05; Figure S3: Quantitative RT-PCR analysis of ALP and Runx2 expression during hBMSCs osteogenic differentiation stimulated with PLGA (*n* = 3 for all groups).
