# Supplementary material for: Enhancing Osteogenic Potential: Controlled Release of Dopamine D1 Receptor Agonist SKF38393 Compared to Free Administration
Source: Biomedicines. 2024 May 9;12(5):1046. doi: 10.3390/biomedicines12051046 (PMC11117781; doi:10.3390/biomedicines12051046)
Supplement: Supplementary file 1 [file biomedicines-12-01046-s001.zip › Figure Legends.pdf]

Figure Legends

Figure 1. Infographic showing the fabrication of PLGA/SKF38393 via the S/O/W method. Created with Biorender.

Figure 2. (**A**) SEM images of PLGA and PLGA/SKF38393. (**B**) Particle size analysis of PLGA and PLGA/SKF38393. (**C**) FT-IR spectra of PLGA, SKF38393, and PLGA/SKF38393. (**D**) Release rate of SKF38393 in PLGA/SKF38393.

Figure 3. (**A**) Fluorescent staining indicating the cell adhesion of PLGA/SKF38393. (**B**) The safe concentration of PLGA/SKF38393 based on a CCK-8 assay. (**C**) Live/dead staining.

Figure 4. ALP staining and relative ALP activity: Alizarin red staining and total absorbance measurements during late hBMSC osteogenic differentiation stimulated with SKF38393 and PLGA/SKF38393 ($n = 3$ for all groups), a: CTL; b: SKF38393; c: PLGA+SKF38393. * $p < 0.05$.

Figure 5. (**A**) Quantitative RT-PCR analysis of ALP and Runx2 expression during hBMSC osteogenic differentiation stimulated with SKF38393 and PLGA/SKF38393 ($n = 3$ for all groups), * $p < 0.05$. (**B**) Western blot analysis of ALP and Runx2 expression during hBMSC osteogenic differentiation stimulated with SKF38393 and PLGA/SKF38393.

Figure 6. Micro-CT images of the ROIs and changes in the BV/TV, Tb.th, Tb.Sp, and BMD of the femurs in all five groups. The parameters are expressed as the mean ± SD, $n = 5$ specimens per group, * $p < 0.05$.

Figure 7. Changes in the histological test for all five groups determined via HE staining and Masson staining.

Figure 8. The q-PCR array results showing the mRNA expression involved in osteogenic differentiation in hBMSCs stimulated with SKF38393 and PLGA/SKF38393.

Figure S1: ROI of Micro-CT

Figure S2: Safety concentration of SKF38393 by CCK-8 assay. * $p < 0.05$.

Figure S3: Quantitative RT-PCR analysis of ALP and Runx2 expression during hBMSCs osteogenic differentiation stimulated with PLGA (n = 3 for all groups)
