# Supplementary figures and images for: Enhancing Osteogenic Potential: Controlled Release of Dopamine D1 Receptor Agonist SKF38393 Compared to Free Administration
Source: Biomedicines. 2024 May 9;12(5):1046. doi: 10.3390/biomedicines12051046 (PMC11117781; doi:10.3390/biomedicines12051046)

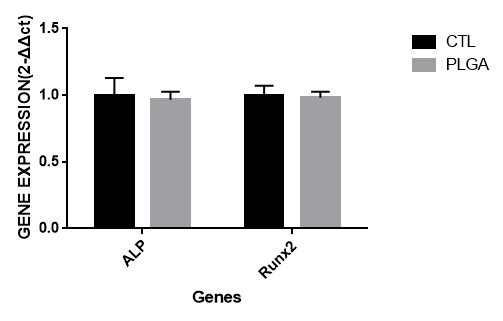

Supplement: Supplementary file 1 [file biomedicines-12-01046-s001.zip › Figure S3.png]
